# Supplementary material for: Mapping the evolution of fertility support policies in China: A content and instrumental analysis
Source: PLoS One. 2025 Oct 9;20(10):e0332137. doi: 10.1371/journal.pone.0332137 (PMC12510515; doi:10.1371/journal.pone.0332137)
Supplement: S1 Appendix — (ZIP) [file pone.0332137.s001.zip › S1 Appendix. 226 original policy documents/154-中华人民共和国劳动保险条例(1953年修正).doc]

中华人民共和国劳动保险条例(1953修正)

**发布部门：** [政务院(已变更)](https://www.pkulaw.com/law/chl?Aggs.IssueDepartment=70202)

**发布日期：**1953.01.02

**实施日期：**1953.01.02

**时效性：** [部分失效](https://www.pkulaw.com/law/chl?Aggs.TimelinessDic=05)

**效力级别：** [行政法规](https://www.pkulaw.com/law/chl?Aggs.EffectivenessDic=XC0201)

**法规类别：** [工资福利与劳动保险](https://www.pkulaw.com/law/chl?Aggs.Category=08707)

**部分失效依据：**本篇法规中“有关女工人、女职员生育待遇的规定”已被[女职工劳动保护规定[失效]](javascript:TMP(3935,0,'chl'))废止

中华人民共和国劳动保险条例
（一九五一年二月二十六日政务院发布 一九五三年一月二日政务院修正发布）

第一章　　总则

　　第一条　为了保护工人职员的健康，减轻其生活中的困难，特依据目前经济条件，制定本条例。

　　第二条　本条例的实施，采取逐步推广办法，目前的实施范围暂定如下：

　　甲、有工人职员一百人以上的国营、公私合营、私营及合作社经营的工厂、矿场及其附属单位；

　　乙、铁路、航运、邮电的各企业单位及附属单位；

　　丙、工、矿、交通事业的基本建设单位；

　　丁、国家建筑公司。

　　关于本条例的实施范围继续推广办法由中央人民政府劳动部根据实际情况随时提出意见，报请中央人民政府政务院决定之。

　　第三条　不实行本条例的企业及季节性的企业，其有关劳动保险事项，得由各该企业或其所属产业或行业的行政方面或资方与工会组织，根据本条例原则及本企业、本产业或本行业的实际情况协商，订立集体合同规定之。

　　第四条　凡在实行劳动保险的企业内工作的工人与职员（包括学徒），不分民族、年龄、性别和国籍，均适用本条例，但被剥夺政治权利者除外。

　　第五条　凡在实行劳动保险的企业内工作的临时工、季节工与试用人员，其劳动保险待遇在本条例实施细则中另行规定之。

　　第六条　本条例适用范围内的企业 ，因经济特殊困难不易维持 ，或尚未正式开工营业者，经企业行政方面或资方与工会基层委员会双方协商同意，并报请当地人民政府劳动行政机关批准后，可暂缓实行本条例。

第二章　　劳动保险金的征集与保管②

　　第七条　本条例所规定之劳动保险的各项费用，全部由实行劳动保险的企业行政方面或资方负担，其中一部分由各企业行政方面或资方直接支付，另一部分由企业行政方面或资方缴纳劳动保险金，交工会组织办理。

　　第八条　凡根据本条例实行劳动保险的企业，其行政方面或资方须按月缴纳相当于各该企业全部工人与职员工资总额的百分之三，作为劳动保险金。此项劳动保险金，不得在工人与职员工资内扣除，并不得向工人与职员另行征收。

　　第九条　劳动保险金的征集与保管方法如下：

　　甲、企业行政方面或资方，须按照上月份工资总额计算，于每月一日至十日限期内，一次向中华全国总工会指定代收劳动保险金的国家银行，缴纳每月应缴的劳动保险金。

　　乙、在开始实行劳动保险的头两个月内，由企业行政方面或资方按月缴纳的劳动保险金，全数存于中华全国总工会户内，作为劳动保险总基金，为举办集体劳动保险事业之用。自开始实行的第三个月起，每月缴纳的劳动保险金，其中百分之三十，存于中华全国总工会户内，作为劳动保险总基金；百分之七十存于各该企业工会基层委员会户内，作为劳动保险基金，为支付工人与职员按照本条例应得的抚恤费、补助费与救济费之用。

　　第十条　企业行政方面或资方逾期未缴或欠缴劳动保险金时，须每日增交滞纳金，其数额为未缴部分百分之一。如逾期二十日尚未缴纳，对于国营、地方国营、公私合营或合作社经营的企业，由工会基层委员会通知当地国家银行从其经费中扣缴；对于私营企业，由工会基层委员会报告当地人民政府劳动行政机关，对该企业资方追究责任。

　　第十一条　劳动保险金的保管，由中华全国总工会委托中国人民银行代理之。

第三章　　各项劳动保险待遇的规定

　　第十二条　因工负伤、残废待遇的规定∶

　　甲、工人与职员因工负伤，应在该企业医疗所、医院或特约医院医治。如该企业医疗所、医院或特约医院无法医治时，应由该企业行政方面或资方转送其他医院医治。其全部诊疗费、药费、住院费、住院时的膳费与就医路费，均由企业行政方面或资方负担。在医疗期间，工资照发。

　　乙、工人与职员因工负伤确定为残废时，按下列情况，由劳动保险基金项下按月付给因工残废抚恤费或因工残废补助费：

　　一、完全丧失劳动力不能工作退职后，饮食起居需人扶助者，其因工残废抚恤

　　费的数额为本人工资百分之七十五，付至死亡时止。

　　二、完全丧失劳动力不能工作退职后，饮食起居不需人扶助者，其因工残废抚恤费的数额为本人工资百分之六十，付至恢复劳动力或死亡时止。劳动力恢复后应由企业行政方面或资方给予适当工作。

　　三、部分丧失劳动力尚能工作者，应由企业行政方面或资方分配适当工作，并由劳动保险基金项下，按其残废后丧失劳动力的程度，付给因工残废补助费，其数额为残废前本人工资的百分之十至三十，但与残废后复工时的工资合计不得超过残废前本人工资。详细办法在实施细则中规定之。

　　丙、工人与职员因工负伤而致残废者，其残废状况的确定与变更，由残废审查委员会审定。详细办法在实施细则中规定之。

　　第十三条　疾病、非因工负伤、残废待遇的规定：

　　甲、工人与职员疾病或非因工负伤，在该企业医疗所、医院、特约医院或特约中西医师处医治时，其所需诊疗费、手术费、住院费及普通药费均由企业行政方面或资方负担；贵重药费、住院的膳费及就医路费由本人负担，如本人经济状况确有困难，得由劳动保险基金项下酌子补助。患病及非因工负伤的工人职员，应否住院或转院医治及出院时间，应完全由医院决定之。

　　乙、工人与职员疾病或非因工负伤停止工作医疗时，其停止工作医疗期间连续在六个月以内者，按其本企业工龄的长短，由该企业行政方面或资方发给病伤假期工资，其数额为本人工资百分之六十至一百；停止工作连续医疗期间在六个月以上时，改由劳动保险基金项下按月付给疾病或非因工负伤救济费，其数额为本人工资百分之四十至百分之六十，至能工作或确定为残废或死亡时止。详细办法在实施细则中规定之。

　　丙、工人与职员因病或非因工负伤医疗终结确定为残废，完全丧失劳动力退职后，病伤假期工资或疾病非因工负伤救济费停发，改由劳动保险基金项下发给非因工残废救济费，其数额按下列情况规定之：饮食起居需人扶助者为本人工资百分之五十，饮食起居不需人扶助者为本人工资百分之四十，至恢复劳动力或死亡时止；部分丧失劳动力尚能工作者不予发给。关于残废状况的确定与变更，适用第十二条丙款的规定。

　　丁、工人与职员疾病或非因工负伤痊愈或非因工残废复劳动力后，经负责医疗机关提出证明，该企业行政方面或资方应给予适当工作。

　　戊、工人与职员供养的直系亲属患病时，得在该企业医疗所、医院、特约医院或特约中西医师处免费诊治，手术费及普通药费，由企业行政方面或资方负担二分之一，贵重药费、就医路费、住院费、住院时的膳费及其他一切费用，均由本人自理。

　　第十四条　工人与职员及其供养的直系亲属死亡时待遇的规定∶

　　甲、工人与职员因工死亡时，由该企业行政方面或资方发给丧葬费，其数额为该企业全部工人与职员平均工资三个月；另由劳动保险基金项下，按其供养的直系亲属人数，每月付给供养直系亲属抚恤费，其数额为死者本人工资百分之二十五至百分之五十，至受供养者失去受供养的条件时为止。详细办法在实施细则中规定之。

　　乙、工人与职员因病或非因工负伤死亡时，由劳动保险基金项下付给丧葬补助费，其数额为该企业全部工人与职员平均工资二个月；另由劳动保险基金项下，按其供养直系亲属人数，付给供养直亲属救济费其数额为死者本人工资六个月到十二个月。详细办法在实施细则中规定之。

　　丙、工人与职员因工负伤致成残废完全丧失劳动力退职后死亡时，应按本条甲款的规定，付给丧葬费及供养直系亲属抚恤费。退职养老后死亡时或非因工残废完全丧失劳动力退职后死亡时，应按本条乙款的规定，付给丧葬补助费及供养直系亲属救济费。

　　丁、工人与职员供养的直系亲属死亡时，由劳动保险基金项下付给供养直系亲属丧葬补助费：死者年龄在十周岁以上者，其数额为该企业全部工人与职员平均工资一个月的二分之一；一周岁至十周岁者，为平均工资一个月的三分之一；不满一周岁者不给。

　　第十五条　养老待遇的规定：

　　甲、男工人与男职员年满六十岁，一般工龄已满二十五年，本企业工龄满五年者，可退职养老。退职后，由劳动保险基金项下，按其本企业工龄的长短，按月付给退职养老补助费，其数额为本人工资的百分之五十至七十，付至死亡时止。合于养老条件，但因该企业工作的需要，留其继续工作者，除发给原有工资外，应由劳动保险基金项下，按其本企业工龄的长短，每月付给在职养老补助费，其数额为本人工资百分之十至二十。详细办法在实施细则中规定之。

　　乙、女工人与女职员年满五十岁，一般工龄满二十年，本企业工龄满五年者，得享受本条甲款规定的养老补助费待遇。

　　丙、井下矿工或固定在华氏三十二度以下的低温的工作场所或华氏一百度以上的高温工作场所工作者，男工人与男职员年满五十五岁，女工人与女职员年满四十五岁，均得享受本条甲款规定的养老补助费待遇。计算其一般工龄及本企业工龄时，每在此种场所工作一年，均作一年零三个月计算。

　　丁、在提炼或制造铅、汞、砒、磷、酸的工业中及其他化学、兵工工业中，直接从事有害身体健康工作者，男工人与男职员年满五十五岁，女工人与女职员年满四十五岁，均得享受本条甲款规定的养老补助费待遇。计算其一般工龄及本企业工龄时，每从事此种工作一年，均作一年零六个月计算。

　　第十六条　生育待遇的规定：

　　甲、女工人与女职员生育，产前产后共给假五十六日，产假期间，工资照发。

　　乙、女工人与女职员怀孕不满七个月小产时，得根据医师的意见给予三十日以内的产假，产假期间，工资照发。

　　丙、女工人与女职员难产或双生时，增给假期十四日，工资照发。

　　丁、女工人与女职员怀孕，在该企业医疗所、医院或特约院检查或分娩时，其检查费与接生费由企业行政方面或资方负担，其他费用均按第十三条甲款的规定处理。

　　戊、产假期间（不论正产或小产）仍不能工作者，经医院证明后，按第十三条关于疾病待遇的规定处理之。

　　己、女工人与女职员或男工人与男职员之妻生育时，由劳动保险基金项下发给生育补助费四万元③。

　　第十七条　集体劳动保险事业的规定：

　　甲、凡在实行劳动保险的各企业内工作的工人与职员，均有享受集体劳动保险事业的权利。详细办法由中华全国总工会制定之。

　　乙、各企业工会基层委员会得根据各该企业的经济情况及工人与职员的需要，与企业行政方面或资方共同办理疗养所、业余疗养所、托儿所等集体劳动保险事业。详细办法在实施细则中规定之。

　　丙、中华全国总工会可举办或委托各地方或各产业工会组织举办下列各项集体劳动保险事业。

　　一、疗养所；

　　二、休养所；

　　三、养老院；

　　四、孤儿保育院；

　　五、残废院；

　　六、其他。

　　第十八条　凡在实行劳动保险企业内工作的工人与职员，未加入工会者，除因工负伤、残废、 死亡待遇， 生育待遇，因病或非因工负伤治疗待遇，供养直系亲属疾病治疗待遇，均得按本条例的规定享受外，其他各项，如疾病或非因工负伤医疗期间的工资与救济费，非因工残废救济费，供养直系亲属救济费，养老补助费，丧葬补助费，只能领取规定额的半数。

第四章　享受优异劳动保险待遇的规定

　　第十九条　凡对本企业有特殊贡献的劳动模范及转入本企业工作的战斗英雄，经工会基层委员会提出，并经各省、市工会组织或产业工会全国委员会的批准，得享受下列较优异的劳动保险待遇：

　　甲、疾病或非因工负伤的贵重药费、就医路费、住院膳费，概由企业行政方面或资方负担。

　　乙、疾病或非因工负伤医疗期间前六个月工资照发。疾病或非因工负伤救济费及非因工残废救济费，一律付给本人工资百分之六十。因工残废抚恤费为本人工资百分之一百。因工残废补助费为残废前本人工资与残废后复工时本人工资的差额。因工死亡供养直系亲属抚恤费为本人工资百分之三十至百分之六十。退职养老补助费为本人工资百分之六十至百分之八十。在职养老补助费为本人工资百分之二十至百分之三十。详细办法在实施细则中规定之。

　　丙、有享受集体劳动保险事业的优先权。

　　第二十条　残废军人转入本企业工作者，疾病或非因工负伤停止工作医疗期间，不计本企业工龄长短，前六个月工资照发，前六个月以后，仍按第十三条乙款规定办理。

第五章　劳动保险金的支配

　　第二十一条　劳动保险金的支配办法如下：

　　甲、劳动保险总基金由中华全国总工会用以举办集体劳动保险事业。

　　乙、劳动保险基金由工会基层委员会用以支付各项抚恤费、补助费与救济费及本企业集体劳动保险事业的补助费。每月结算一次，其余额全部转入省、市工会组织或产业工会全国委员会户内，作为劳动保险调剂金（以下简称调剂金）。

　　丙、调剂金由省、市工会组织或产业工会全国委员会用于对所属各工会基层委员会劳动保险基金不足开支时的补助或举办集体劳动保险事业之用。各产业工会全国委员会得授权其地方机构，掌管调剂金的调用。中华全国总工会对所属各省、市工会组织，各产业工会全国委员会的调剂金，有统筹调用之权，并得用以举办集体劳动保险事业。如省、市工会组织或产业工会全国委员会调剂金不足开支，得申请中华全国总工会调拨调剂金补助之。

　　第二十二条　劳动保险金，除用于劳动保险事业外，不得移作其他用途。

　　第二十三条　各企业的会计部门，均须设立劳动保险基金的独立会计，负责办理劳动保险基金的收支事宜。劳动保险基金会计制度，由中央人民政府劳动部会同中华全国总工会制定之。

　　第二十四条　劳动保险调剂金的收支事宜，由各级工会组织的财务部门根据中华全国总工会的规定办理之。

第六章　劳动保险事业的执行与监督

　　第二十五条　各工会基层委员会，为执行劳动保险业务的基层单位，其主要工作为：督促劳动保险金的缴纳；决定劳动保险基金的支付；监督本条例所规定由企业行政方面或资方直接支付的各项费用的开支；推动该企业改进集体劳动保险事业及医疗卫生工作；执行一切有关劳动保险的实际业务；每月编造劳动保险基金月报表，每年编造预算、决算、业务计划书及业务报告书，报告省、市工会组织和产业工会全国委员会及当地人民政府劳动行政机关；并向工会全体会员大会或代表大会报告工作。

　　第二十六条　各工会基层委员会的经费审查委员会，应按月审核劳动保险基金收支账目及本条例所规定的由企业行政方面或资方直接支付的各项费用，并公布之。

　　第二十七条　各省、市工会组织，各产业工会全国委员会或地区委员会对所属各工会基层委员会的劳动保险业务，负指导督促之责，审核劳动保险基金的收支月报表、决算、决算及劳动保险基金的收支有无错误，接受工人与职员有关劳动保险事件的申诉，每月编造劳动保险基金及调剂金的收支月报表，每年编造预算、决算、业务计划书及业务告书，并依下列程序报告：

　　甲、各省、市工会组织向当地人民政府劳动行政机关及大行政区工会组织报告。

　　乙、各产业工会全国委员会向中华全国总工会及中央人民政府劳动部报告。

　　第二十八条　各大行政区工会组织对所属各省、市工会组织及其区域内产业工会组织的劳动保险工作， 负指导督促之责， 审核省、市工会组织劳动保险基金及调剂金的收支月报表、预算、决算、业务计划书及业务报告书，并每三个月编造劳动保险基金收支报告，每年编造预算、决算、业务计划书及业务报告书，报告所在地大行政区人民政府劳动部、中央人民政府劳动部及中华全国总工会。

　　第二十九条　中华全国总工会为全国劳动保险事业的最高领导机关，统筹全国劳动保险事业的进行，督导所属各地方工会组织，各产业工会组织有关劳动保险事业的执行；审核并汇编劳动保险基金的收支报告表，每年编造劳动保险金的预算、决算、业务计划书及业务报告书，并送中央人民政府劳动部、财政部备查。

　　第三十条　各级人民政府劳动行政机关应监督劳动保险金的缴纳，检查劳动保险业务的执行，并处理有关劳动保险事件的申诉。

　　第三十一条　中央人民政府劳动部为全国劳动保险业务的最高监督机关，贯彻[劳动保险条例](https://www.pkulaw.com/chl/55d00ceb2d6f9d82bdfb.html#class=alink)的实施，检查全国劳动保险业务的执行，其检查制度另订之。

第七章　附则

　　第三十二条　本条例由中央人民政府政务院通过后发布施行，修改时同。

　　注：①1．有关在劳动保险基金项下支付的各项费用，根据一九七三年五月十五日财政部财企字第41号文的规定，改在营业外支付；

　　2．有关医疗待遇的规定，与劳动部、全国总工会一九六六年四月十五日《关于改进职工劳保医疗制度几个问题的通知》的规定有抵触时，均按一九六六年通知的规定执行；

　　3．有关养老待遇和因工、非因工残废完全丧失劳动能力的待遇的规定，与全国人民代表大会常务委员会原则批准、一九七八年六月二日国务院发布的《国务院关于安置老弱病残干部的暂行办法》和《国务院关于工人退休、退职的暂行办法》中的规定有抵触时，应按一九七八年两个暂行办法的规定执行。

　　②劳动保险金的征集管理已于一九六九年起停止执行。

　　③系指旧人民币，折合新人民币四元。
